# Role of cassava CC-type glutaredoxin *MeGRXC3* in regulating sensitivity to mannitol-induced osmotic stress dependent on its nuclear activity

**DOI:** 10.1186/s12870-022-03433-y

**Published:** 2022-01-20

**Authors:** Meng-Bin Ruan, Xiao-Ling Yu, Xin Guo, Ping-Juan Zhao, Ming Peng

**Affiliations:** 1 Institute of Tropical Bioscience and Biotechnology, Chinese Academy of Tropical Agricultural Sciences, Haikou, 571101 China; 2 Key Laboratory of Biology and Genetic Resources of Torpical Crops, Ministry of Agriculture, Haikou, 571101 China; 3 Huazhong Agricultural University, Wuhan, 430070 China

**Keywords:** Cassava (*Manihot esculenta*), CC-type glutaredoxin, Nuclear activity, Mannitol-induced osmotic stress

## Abstract

**Background:**

We previously identified six drought-inducible CC-type glutaredoxins in cassava cultivars, however, less is known about their potential role in the molecular mechanism by which cassava adapted to abiotic stress.

**Results:**

Herein, we investigate one of cassava drought-responsive CC-type glutaredoxins, namely *MeGRXC3*, that involved in regulation of mannitol-induced inhibition on seed germination and seedling growth in transgenic *Arabidopsis*. *MeGRXC3* overexpression up-regulates several stress-related transcription factor genes, such as *PDF1.2*, *ERF6*, *ORA59, DREB2A*, *WRKY40*, and *WRKY53* in *Arabidopsis*. Protein interaction assays show that MeGRXC3 interacts with *Arabidopsis* TGA2 and TGA5 in the nucleus. Eliminated nuclear localization of MeGRXC3 failed to result mannitol-induced inhibition of seed germination and seedling growth in transgenic *Arabidopsis.* Mutation analysis of MeGRXC3 indicates the importance of conserved motifs for its transactivation activity in yeast. Additionally, these motifs are also indispensable for its functionality in regulating mannitol-induced inhibition of seed germination and enhancement of the stress-related transcription factors in transgenic *Arabidopsis*.

**Conclusions:**

*MeGRXC3* overexpression confers mannitol sensitivity in transgenic *Arabidopsis* possibly through interaction with TGA2/5 in the nucleus, and nuclear activity of MeGRXC3 is required for its function.

**Supplementary Information:**

The online version contains supplementary material available at 10.1186/s12870-022-03433-y.

## Background

Reactive oxygen species (ROS) have been considered harmful to plant cells; however, they are also playing signaling roles in plant response to stress [1]. Glutaredoxin (GRX) is essential for redox homeostasis and ROS signalling in plant cells [2]. GRXs are in particular studied for their involvement in oxidative stress responses [2–4]. GRXs are classified into five subgroups, and CC-type GRXs are members of a land plant specific GRX subgroup that was characterized as ROXY family in *Arabidopsis* [2]. There 21 CC-type GRXs were in *Arabidopsis* and maize [5, 6], whereas 17 were identified in rice [6, 7] and 18 were identified in cassava [8]. Comparative analysis of evolutionary informative plant species indicated that CC-type GRXs number expanded and might gain new functions during land plant evolution [6, 7, 9]. The functions and the molecular mechanism of CC-type GRXs in plants remain largely unknown, especially in cassava, an important tropical tuber crop.

Although *ROXY1*, the first reported CC-type GRX regulates petal development, they are also involved in plant cell ROS homeostasis under both biotic and abiotic stress [10–12]. Overexpression of the *ROXY1* strongly increased ROS accumulation and caused higher susceptibility to botrytis in *Arabidopsis* [6]. On the other hand, La Camera et al. [13] showed that the mutant of *GRXS13*/*ROXY18* possessed increased resistance to botrytis. The *roxy18/grxs13* mutant showed a higher basal and photo-oxidative stress induced ROS accumulation and therefore caused sensitivity to methyl viologen (MV) and high light (HL), while overexpression of *ROXY18/GRXS13* resulted lower ROS accumulation under MV and HL treatments [14]. These results indicate that CC-type GRXs may play antagonistic roles in ROS homeostasis.

Several CC-type GRXs have shown their potential roles in regulating abiotic stress tolerance. Genetic variation in *ZmGRXCC14* shows significant association with drought tolerance at seedling stage [5]. Expression of *OsGRX6* changes depending on the level of available nitrate, overexpression of this gene delayed leaf senescence in rice [15]. The expression of *OsGRX8* could be induced by auxin and abiotic stresses [16]. Overexpression of *OsGRX8* enhanced tolerance to various abiotic stresses such as salinity, osmotic and oxidative stress in transgenic *Arabidopsis*, while repression of *OsGRX8* by RNAi in rice caused a dramatically seed germination inhibition under mannitol treatment [16]. A rice CC-type GRX, *OsGRX_C7* plays a positive response in salt induced stress by regulating the expression of transports engaged in Na^+^ homeostasis [17]. Moreover, *OsGRX_C7* is also involving in arsenic tolerance by altering the transcript of *NIPs* [18, 19]. Most CC-type GRXs play positively regulator role on abiotic stress tolerance in different plants, on the contrary, cassava CC-type GRX *MeGRXC15* negatively regulates drought tolerance in transgenic *Arabidopsis* [8]. It needs more efforts to unravel functions and molecular mechanisms of cassava CC-type GRXs.Yeast-two-hybrid assay showed that several *Arabidopsis* CC-type GRXs were able to interact with the bZIP transcription factor TGACG-BINDING FACTOR 2 (TGA2) [20, 21]. They play regulatory roles by post-translationally modifying TGA transcription factors in either negative or positive means. For example, *ROXY1* regulates petal development by negatively modifying a floral specific TGA transcription factor PAN and positively modifying other TGA transcription factors [12]. ROXY19/GRX480 negatively regulates *PDF1.2* and detoxification genes by interaction with TGA2, TGA5, and TGA6 [21, 22]. However, a cassava CC-type GRX *MeGRXC15* interacted with TGA5, function as a positive regulator of several stress-related transcription factors in transgenic *Arabidopsis* [8]. *ROXY8* and *ROXY9* were identified as a regulator in hyponastic growth of *Arabidopsis* by negatively modifying TGA1 and TGA4 [23]. *GRXS25* could trigger metabolism of pesticide residue in tomato plants through activating TGA2 factor by posttranslational redox modification [24]. The interaction between ROXYs and TGA transcription factors dependent on a functionally important conserved amino acid motif, namely ALWL motif at the very C-terminus of ROXYs [21].

Previously works showed that CC-type GRXs are involved in phytohormone signalling pathway by interaction with TGA transcription factors in plants. The *ROXY19/GRX480* expression is induced by salicylic acid (SA), and act as a negative regulator in Jasmonic acid (JA)/Ethylene (ET) pathway [21, 25], suggesting CC-type GRXs regulates crosstalk between SA and JA/ET pathway. The *MeGRXC3* expression is induced by ABA in cassava and regulates several genes which involve in ABA and JA/ET pathway [8], indicating CC-type GRXs also regulates crosstalk between ABA and JA/ET. Overexpression of a rice CC-type GRX *OsGRX6* caused endogenous gibberellin acid (GA) increasing [15]. Moreover, another CC-type GRX namely *PHS9* regulated seed germination of rice through the integration of ROS signaling and ABA signaling [26]. *ROXY8*, *ROXY9*, and *ROXY19/GRX480* involve in auxin pathway by regulating auxin-induced and growth-related genes therefore affect hyponastic growth of *Arabidopsis* [23]. Recently, a tomato CC-type GRX *GRXS25* was identified as a regulator in brassinosteroid (BR) pathway [24]. It seemed likely that CC-type GRXs play numerous roles in plant phytohormone signalling.

Previously, we have identified six drought-inducible CC-type GRXs from two cassava cultivars [8]. In this study, we characterized one of these cassava genes to investigate the potential function of them. We found that four cassava drought-responsive CC-type GRXs, including *MeGRXC3*, *MeGRXC7*, *MeGRXC15*, and *MeGRXC17* showed transcriptional activation ability in yeast. We produced *MeGRXC3, MeGRXC4, MeGRXC15*, and *MeGRXC18* overexpressed transgenic *Arabidopsis*. Only *MeGRXC3* overexpression caused hypersensitivity to mannitol on seed germination and seedling growth in transgenic *Arabidopsis*. In addition, expression of several stress-related transcription factors, including *PDF1.2, ERF1, ERF6, ORA59, DREB2A, WRKY33, WRKY40*, and *WRKY53* was dramatically up-regulated by *MeGRXC3* overexpression in *Arabidopsis*. We also identified two *Arabidopsis* TGA transcription factors, TGA2 and TGA5 that interacted with MeGRXC3 in the nucleus. Further analysis indicates that nuclear activity is required for the function of MeGRXC3 in transgenic *Arabidopsis*. Mutation of conserved motifs in the nuclear localization restricted MeGRXC3 promoted recovery of seed germination from mannitol treatments and dramatically affected its regulation on the expression of stress-related transcription factor in transgenic *Arabidopsis*.

## Results

### MeGRXC3 has transcriptional activation ability in yeast and involved in mannitol-induced stress response in transgenic *Arabidopsis*

**Fig. 1:**
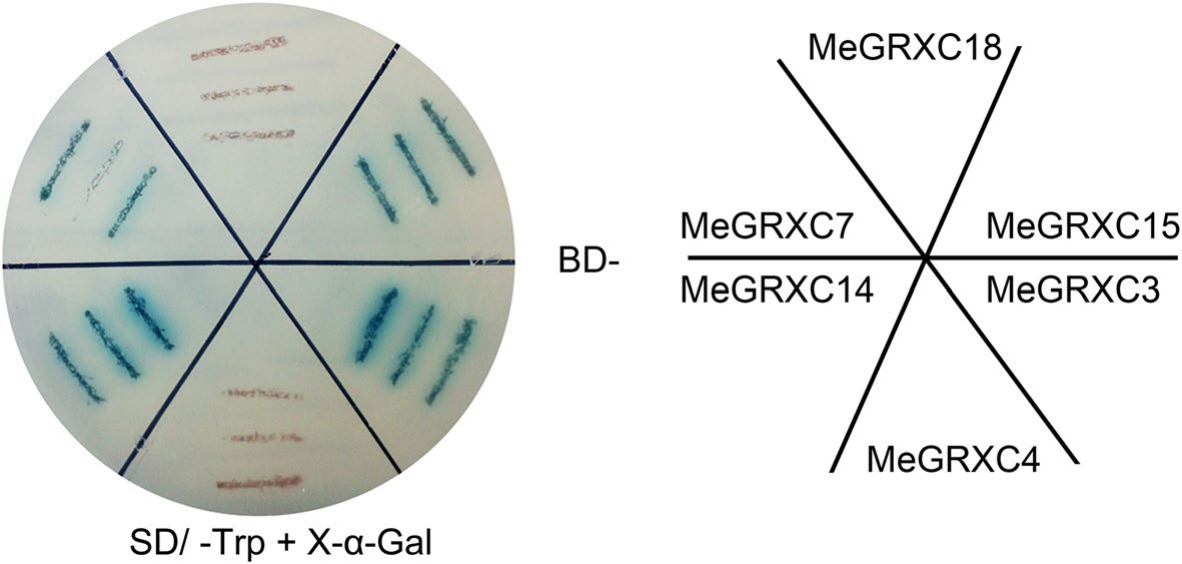
Autonomous transactivation analysis of MeGRXC3, C4, C7, C14, C15 and C18 in yeast. BD indicate GAL4 binding domain

We have previously identified six CC-type GRX genes, *MeGRXC3, MeGRXC4, MeGRXC7, MeGRXC14, MeGRXC15*, and *MeGRXC18* responded to drought in leaves of two cassava cultivars [8]. All these six genes were fused to the GAL4 DNA-binding domain (BD) in *pGBKT7* (Clontech) respectively, and transformed the constructs into yeast Y187 (Clontech). Yeast cells harboring *MeGRXC3*:*pGBKT7*, *MeGRXC7*:*pGBKT7*, *MeGRXC14*:*pGBKT7* and *MeGRXC15*:*pGBKT7* activated X-α-gal activity on SD/ -Trp /X-α-gal medium (Fig. 1), suggesting that MeGRXC3, MeGRXC7, MeGRXC14, and MeGRXC15 has transcriptional activation ability.

**Fig. 2:**
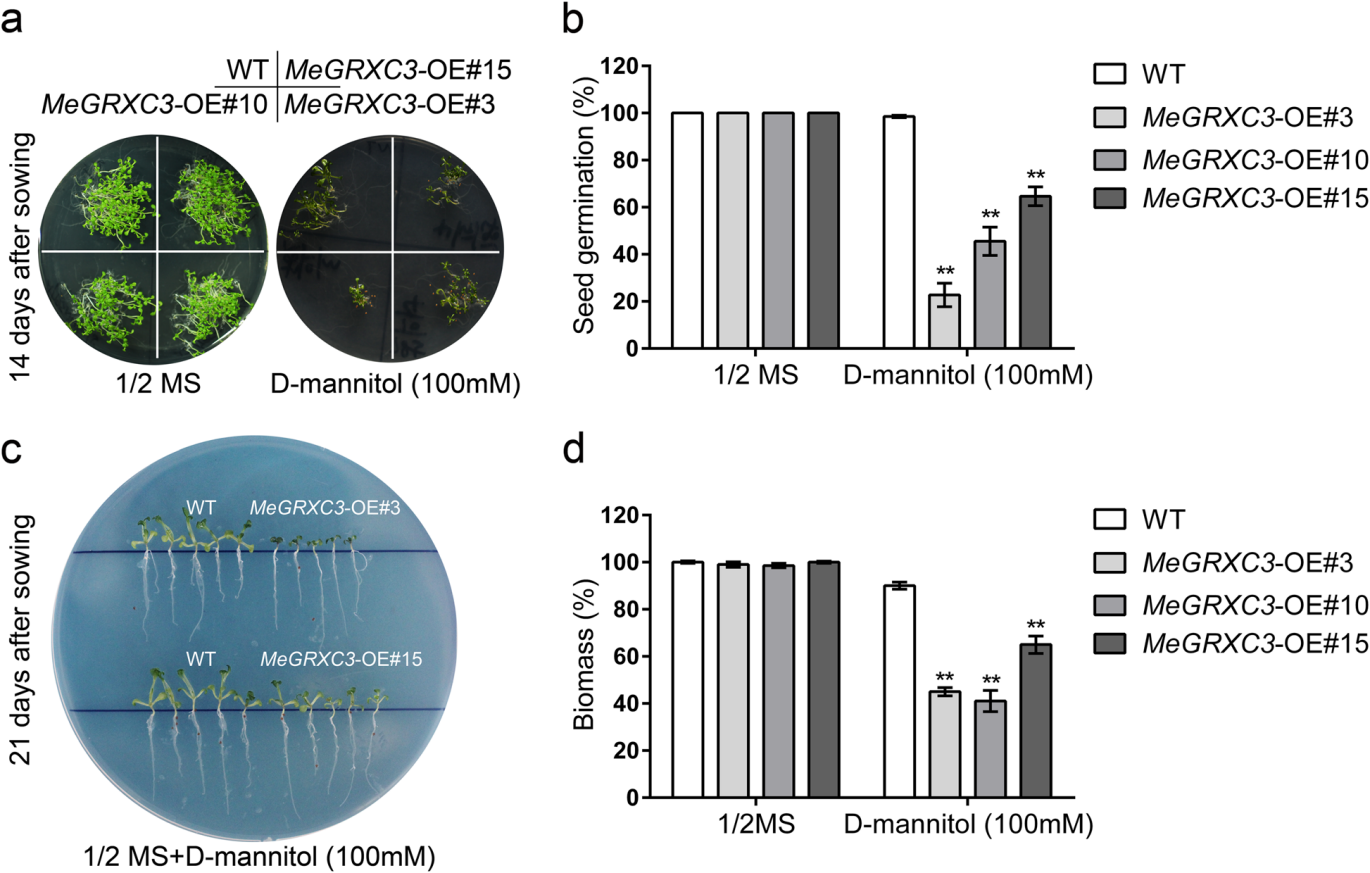
Overexpression of *MeGRXC3* confers mannitol sensitivity in transgenic *Arabidopsis*. (**a**) Seed germination assay of *MeGRXC3*-OE transgenic *Arabidopsis.* Seeds of three independent homozygote lines sown on 1/2 MS medium supplemented with 0 mM or 100 mM D-mannitol respectively, incubated at 22 °C for 14 days. (**b**) Effects of mannitol stress on germination rates. Error bars indicate mean ± SD (*n* = 3). ** *p* ≤ 0.01 (*Student’s* t-test). (**c**) Post-germinated seedling development assay of *MeGRXC3*-OE transgenic *Arabidopsis*. Seedlings grown on 1/2 MS medium were transferred to 1/2 MS supplemented with 0 mM or 100 mM D-mannitol at 7 days after sowing respectively, then incubated at 22 °C for 14 days. (**d**) Effects of mannitol stress on seedling biomass. Error bars indicate mean ± SD (*n* = 5). ** *p* ≤ 0.01 (*Student’s* t-test)

As transgenic work in cassava is extremely difficult and time-consuming, it was impossible to perform large scale functional identification of drought-responsive genes using transgenic cassava. However, *Arabidopsis* could be used as model plant for heterologous expression of drought induced cassava genes in gain of function analysis [27, 28]. Therefore, we produced transgenic *Arabidopsis* that over-expressed *MeGRXC3, MeGRXC4, MeGRXC15*, and *MeGRXC18* respectively (Fig. S1). We selected three homozygous lines for each transgene that exhibited markedly enhanced expression of the CC-type *GRX* in normal conditions for phenotype analyses (Fig. S1). To analyze the abiotic stress tolerance of transgenic *Arabidopsis*, it is commonly to use in vitro setups in which different growth inhibitory compounds are added to the growth medium. Since CC-type GRX may involve in osmotic induced inhibition on seed germination [16], here, we used mannitol, a frequently applied compound to induced osmotic stress in transgenic *Arabidopsis* that overexpressing *MeGRXC3, MeGRXC4, MeGRXC15*, and *MeGRXC18* respectively. We found that 100 mM or 200 mM mannitol treatment severely inhibited seed germination of *MeGRXC3*-OE *Arabidopsis* (Fig. 2a, Fig. S2). However, seed germination of *MeGRXC4*-OE, *MeGRXC15*-OE, and *MeGRXC18*-OE *Arabidopsis* lines is similar to that of wild type when treated with 100 mM or 200 mM mannitol (Fig. S2). These results indicate that *MeGRXC3* may involve in mannitol-induced stress response in transgenic *Arabidopsis*. 100 mM mannitol treatment dramatically reduced seed germination rate of *MeGRXC3*-OE transgenic *Arabidopsis*. Consequently, we used this concentration for subsequent mannitol treatments on transgenic *Arabidopsis*.

### Overexpression of *MeGRXC3* negatively affects seed germination and seedling growth under mannitol-induced stress

As MeGRXC3 shows transcriptional activation ability in yeast and overexpression of *MeGRXC3* caused mannitol-induced inhibition to seed germination in transgenic *Arabidopsis* (Table 1), we selected this gene for further functional analysis. Three *MeGRXC3*-OE *Arabidopsis* lines, *MeGRXC3*-OE#3, #10, #15 were used for further phenotypic assays. Seeds were sown on 1/2 MS medium containing with 0 mM and 100 mM mannitol respectively. Effect of mannitol-induced inhibition to seed germination of transgenic *Arabidopsis* is visible at 14 days after sowing (Fig. 2a). The seed germination rate on 100 mM mannitol was reduced to less than 64.7% in *MeGRXC3*-OE lines and to 98.5% in wild type (Fig. 2b). Thus, seed germination of *MeGRXC3*-OE lines is hypersensitivity to mannitol, suggesting that *MeGRXC3* plays a role in seed germination regulation under mannitol-induced osmotic stress conditions.

**Table 1:** Functional characterization of six cassava drought-responsive CC-type glutaredoxins

Gene name	Transcriptional activation activity ^a^	Sensitivity for seed germination to D-Mannitol ^b^
*MeGRXC3*	+	+
*MeGRXC4*	–	–
*MeGRXC7*	+	n/a
*MeGRXC14*	+	n/a
*MeGRXC15*	+	–
*MeGRXC18*	–	–

^a^Transcriptional activation activity analysis was performed by using GAL4BD-MeGRX fusions in yeast Y187

^b^Seeds of three independent homozygote lines of each *MeGRX* overexpression *Arabidopsis* were incubated on 1/2 MS medium containing with 100 mM D-mannitol for 14 days

To explore whether *MeGRXC3* is involved in mannitol-induced growth inhibition in transgenic *Arabidopsis*, we performed analysis on seedling growth of *MeGRXC3*-OE lines under in vitro stress conditions mediated by 100 mM mannitol (Fig. 2c, Fig. S3). Five-day old seedlings of wild type and transgenic *Arabidopsis* lines were grown on 1/2 MS medium supplement with 100 mM mannitol. Effect of mannitol-induced inhibition to seedling growth is visible after treated by 100 mM mannitol for 14 days (Fig. 2c, Fig. S3). Treatments with 100 mM mannitol reduced 10.1% biomass of wild type seedlings. However, biomass of *MeGRXC3*-OE seedlings was reduced by 35.4 to 59.2% under 100 mM mannitol (Fig. 2d). It can be concluded that *MeGRXC3* overexpression enhanced mannitol-induced growth inhibition in transgenic *Arabidopsis*.

### *MeGRXC3* transgenic regulates expression of several stress related transcription factor genes in *Arabidopsis*

**Fig. 3:**
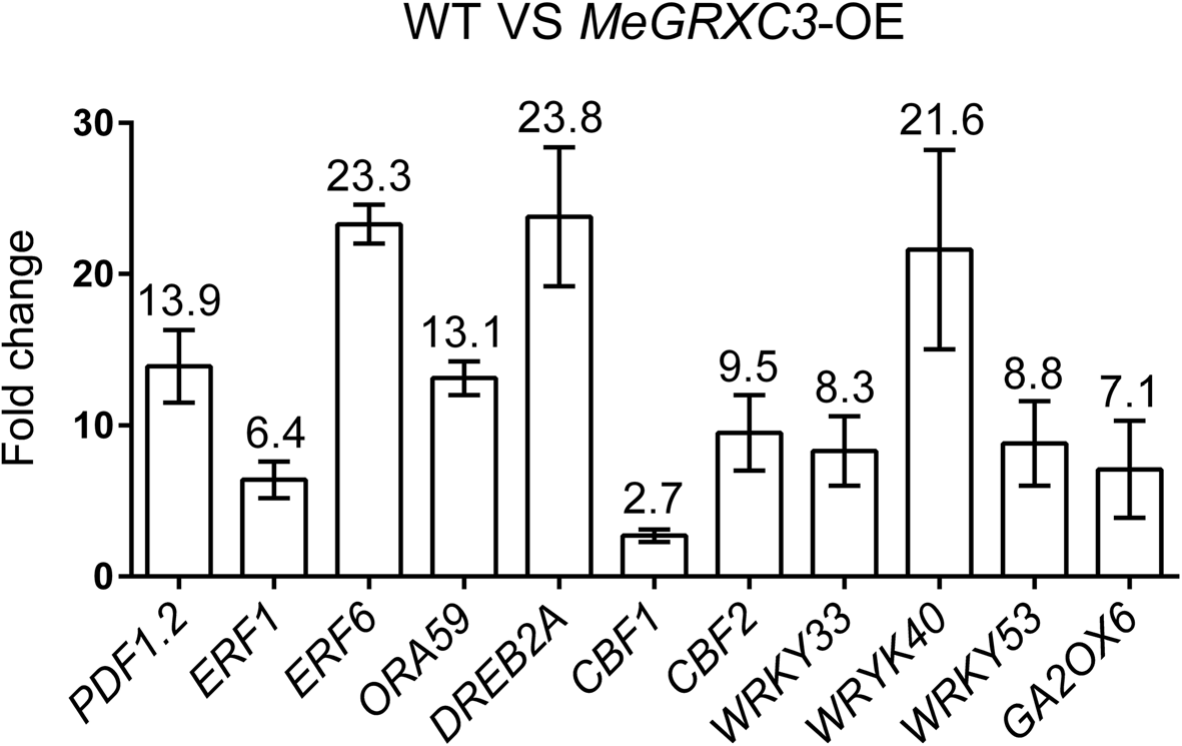
Overexpression of *MeGRXC3* up-regulates several stress-related transcription factor genes in transgenic *Arabidopsis*. Expression levels of selected genes were normalized against wild type *Arabidopsis* (Col-0). Number means fold change of indicating gene. Error bars indicate mean ± SD (*n* = 3)

The CC-type GRXs could suppress *ORA59* promoter activity by interaction with TGA transcription factors in *Arabidopsis* [21], suggesting their gene expression regulation roles in plant. Our previously work also indicated that cassava *MeGRXC15* could regulate several stress-related genes expression in transgenic *Arabidopsis* [8]. Here, to understand the effects of *MeGRXC3* overexpression on gene expression regulation, we performed qPCR assays on *MeGRXC3*-OE *Arabidopsis*. According to the confirmed or proposed roles of plant GRXs [9], and reported mannitol-induced growth inhibition related genes [29], we selected several stress-related genes (*PDF1.2, ERF1, ERF6, ORA59, DREB2A, WRKY33, WRKY40, WRKY53, GA2OX6*) as candidate genes in this study. The qPCR results show that *MeGRXC3* overexpression enhanced the expression of all these selected stress-related genes in transgenic *Arabidopsis* under normal conditions (Fig. 3). Obviously, *MeGRXC3* overexpression dramatically up-regulated expression of *ERF6* (more than 23 folds of wild type), which regulates mannitol-induced growth inhibition in *Arabidopsis* [29]. This suggests that *MeGRXC3* affect mannitol stress tolerance in transgenic *Arabidopsis* probably depends on regulating *ERF6* expression.

### MeGRXC3 interacts with *Arabidopsis* TGA2 and TGA5 in the nucleus

**Fig. 4:**
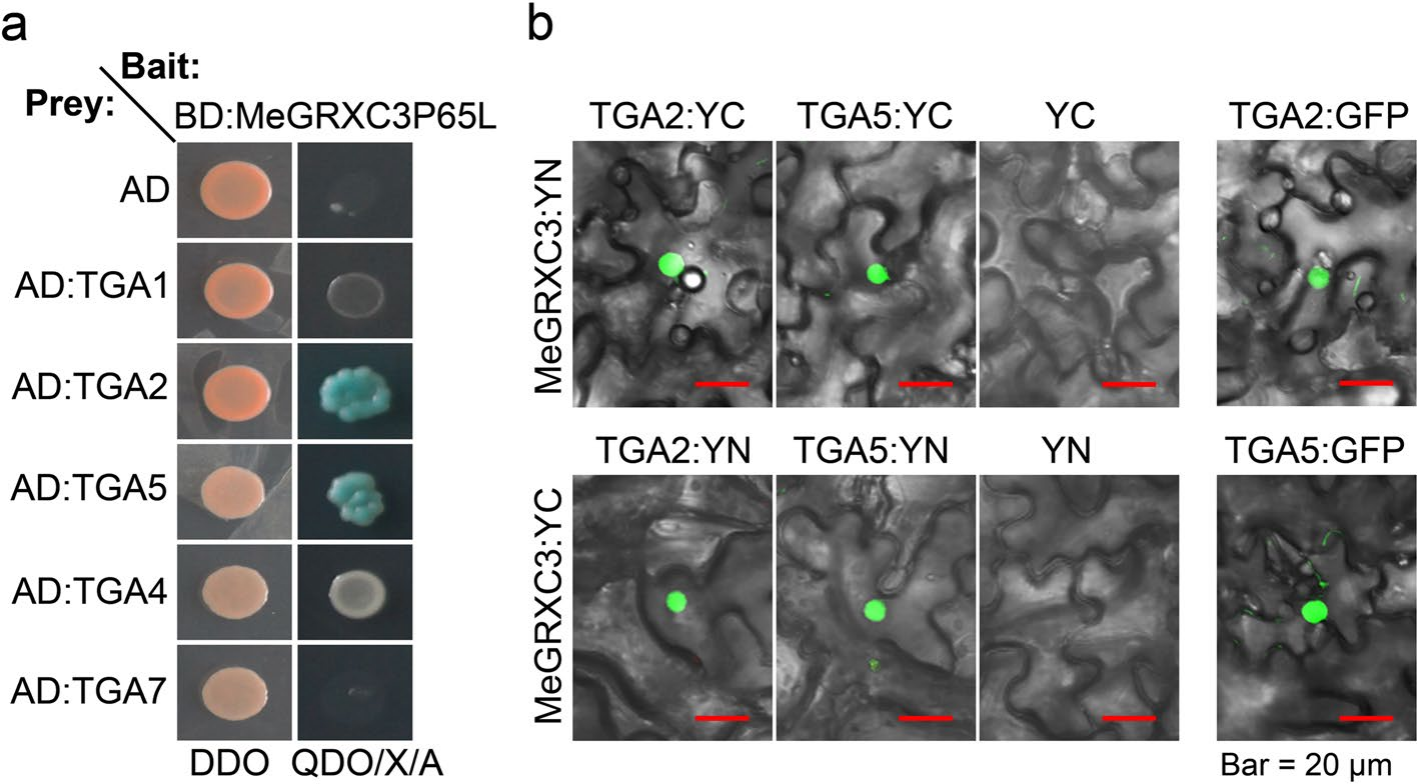
MeGRXC3 interacts with *Arabidopsis* TGA2 and TGA5 in the nucleus. (**a**) Identification of the interaction between MeGRXC3P65L and four TGA factors from *Arabidopsis* by yeast two-hybrid assay. DDO: SD/−Leu/−Trp, QDO/X/A: SD/−Ade/−His/−Leu/−Trp with X-alpha-Gal and Aureobasidin A. (**b**) Bimolecular fluorescence complementation assay of the interaction between MeGRXC3 and TGA2, TGA5 in transiently transformed *N. benthaminan* leaves. Green fluorescence in the nucleus was detected for interactions of MeGRXC3 with TGA2 or TGA5, respectively. As a negative control, co-expression of MeGRXC3:YN/YC with non-fused YC/YN failed to reconstitute a fluorescent YFP chromophore. Green fluorescence in the nucleus was detected for TGA2:GFP and TGA5:GFP as positive controls

Since ROXYs could regulate nuclear gene expression through its interaction with TGA factors [11, 12, 21, 25, 30, 31]. We found that MeGRXC15 could interact with *Arabidopsis* TGA5 or cassava MeTGA074 in the nucleus [8]. To identify target TGA transcription factor that interact with MeGRXC3, yeast two-hybrid assays was conducted using MeGRXC3 as bait to isolate interaction partners from these TGA factors. The results showed that MeGRXC3 protein was able to interact differentially with TGA factors. It showed a strong affinity for TGA2 and TGA5, but no affinity for TGA1, TGA4, and TGA7, respectively (Fig. 4a).

To further investigate the interactions of MeGRXC3 with TGA factors in planta, the BiFC technique was employed. Nuclear green fluorescence was detected for co-expression of MeGRXC3 and TGA2, or TGA5 (Fig. 4b). As negative controls, co-expression of non-fused YN with one of the YC fusion proteins or non-fused YC with one of the YN fusion proteins failed to reconstitute a fluorescent YFP chromophore (Fig. 4b). As positive controls, green fluorescent protein (GFP) was tagged to the C terminus of TGA factors respectively. Green fluorescence was detected only in the nucleus for transiently expression of TGA2: GFP and TGA5: GFP in tobacco (Fig. 4b). This result suggests the possibility of MeGRXC3 in regulating nuclear gene expression via interaction with TGA factors.

### Nucleus localization is required for MeGRXC3 regulating mannitol-induced stress tolerance in transgenic *Arabidopsis*

**Fig. 5:**
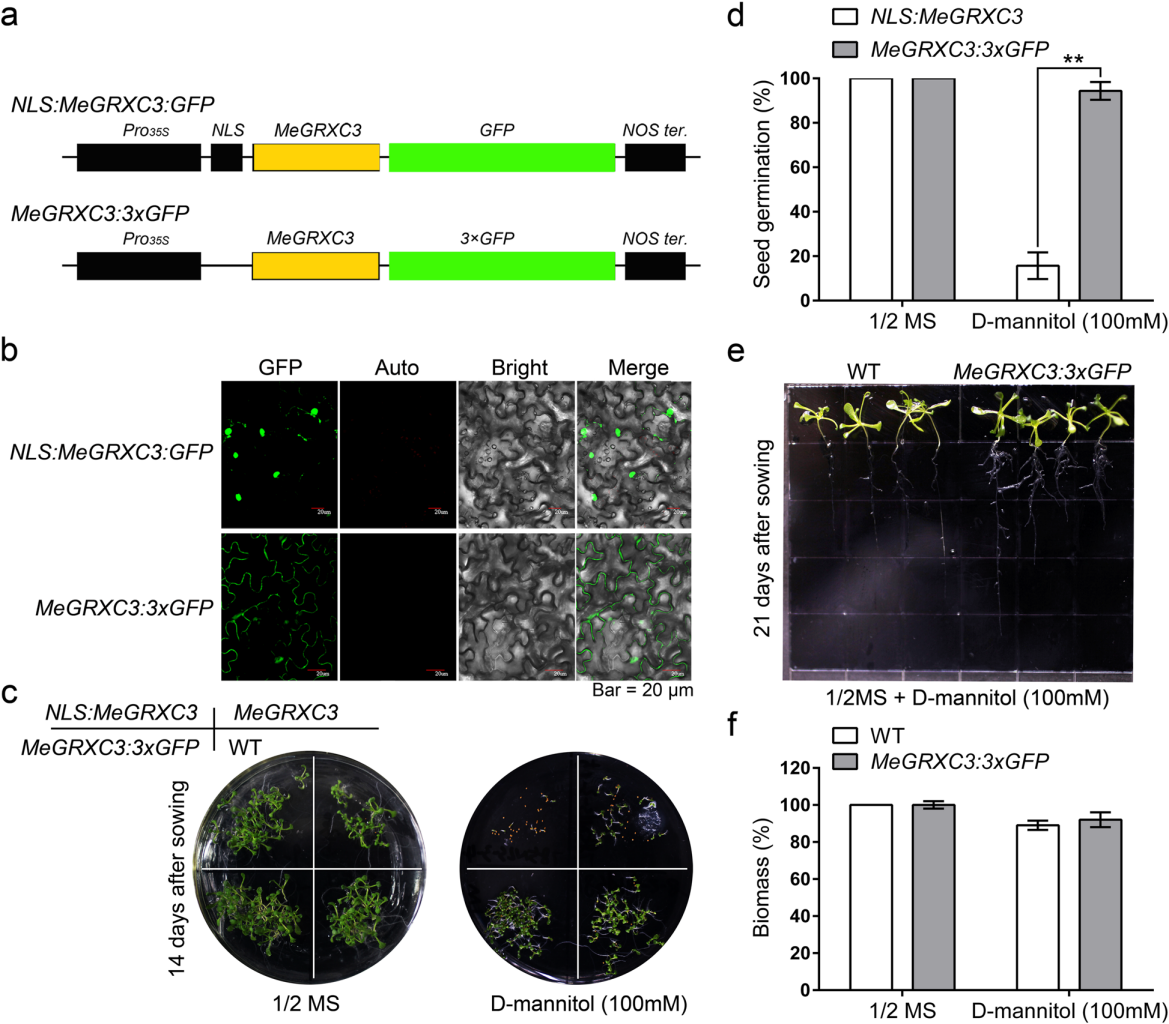
Nuclear activity is required for function of *MeGRXC3* in transgenic *Arabidopsis*. (**a**) Schematic diagram represents DNA constructs of *NLS:MeGRXC3:GFP* and *MeGRXC3:3 × GFP*. NLS, SV40 T large antigen nuclear localization sequence. (**b**) Subcellular localization of NLS:MeGRXC3:GFP and MeGRXC3:3 × GFP fusion proteins in transgenic *Arabidopsis*. (**c**) Seed germination assay of *NLS:MeGRXC3:GFP* and *MeGRXC3:3 × GFP* transgenic *Arabidopsis.* Seeds of three independent homozygote lines sown on 1/2 MS medium supplemented with 0 mM, or 100 mM D-mannitol respectively, incubated at 22 °C for 14 days. (**d**) Effects of mannitol stress on germination rates. Error bars indicate mean ± SD (n = 3). ** *p* ≤ 0.01 (*Student’s* t-test). (**e**) Post-germinated seedling development assay of *NLS:MeGRXC3:GFP* and *MeGRXC3:3 × GFP* transgenic *Arabidopsis*. Seedlings grown on 1/2 MS medium were transferred to 1/2 MS supplemented with 0 mM or 100 mM D-mannitol at 7 days after sowing respectively, then incubated at 22 °C for 14 days. (**f**) Effects of mannitol stress on seedling biomass. Error bars indicate mean ± SD (*n* = 5)

The MeGRXC3:GFP fusion protein shows nucleocytoplasmic distribution in *Arabidopsis* [8]. And BiFC assay show that MeGRXC3 interact with TGA2 and TGA5 in the nucleus. To evaluate whether the nuclear localization is required for function of MeGRXC3 in *Arabidopsis*, we generated fusion proteins of MeGRXC3 that are either excluded from the nucleus and accumulate in the cytoplasm or only localized in the nucleus (Fig. 5a). Exclusive localization of MeGRXC3 protein in the cytoplasm was achieved by cloning three GFP fragments (3 × GFP) in-frame downstream of MeGRXC3, generating a *MeGRXC3:3 × GFP*. Moreover, a nuclear-localized version of MeGRXC3 is created by fusing the nuclear localization signal (NLS) derived from the SV40 large T antigen to the N-terminus of MeGRXC3:GFP, as previously reported for ROXY1 (Li et al., 2009b). We overexpressed these two modified DNA constructs in *Arabidopsis* under the control of the *CaMV 35S* promoter for further analyses (Fig. 5b). Indeed, nuclear localization of MeGRXC3 enhanced seed germination sensitivity to mannitol (Fig. 5c; Fig. S4), which evidenced by less than 15.7% seeds of *NLS:MeGRXC3* lines were germinated under 100 mM mannitol treatment (Fig. 5d). On the contrary, the restricted localization to the cytoplasm disturbed the mannitol sensitivity of seed germination (Fig. 5c, d; Fig. S4). Moreover, overexpression of *MeGRXC3:3 × GFP* did not enhance mannitol-induced growth inhibition in transgenic *Arabidopsis* (Fig. 5e; Fig. S5), as indicated by reduced biomass of *MeGRXC3:3 × GFP* transgenic lines is similar to that of control under 100 mM mannitol treatment (Fig. 5f). These results suggest that nuclear activity of the MeGRXC3 is required and sufficient to regulate response to mannitol-induced osmotic stress in *Arabidopsis*.

### Conserved motifs are required for MeGRXC3 transcriptional activation ability in yeast

**Fig. 6:**
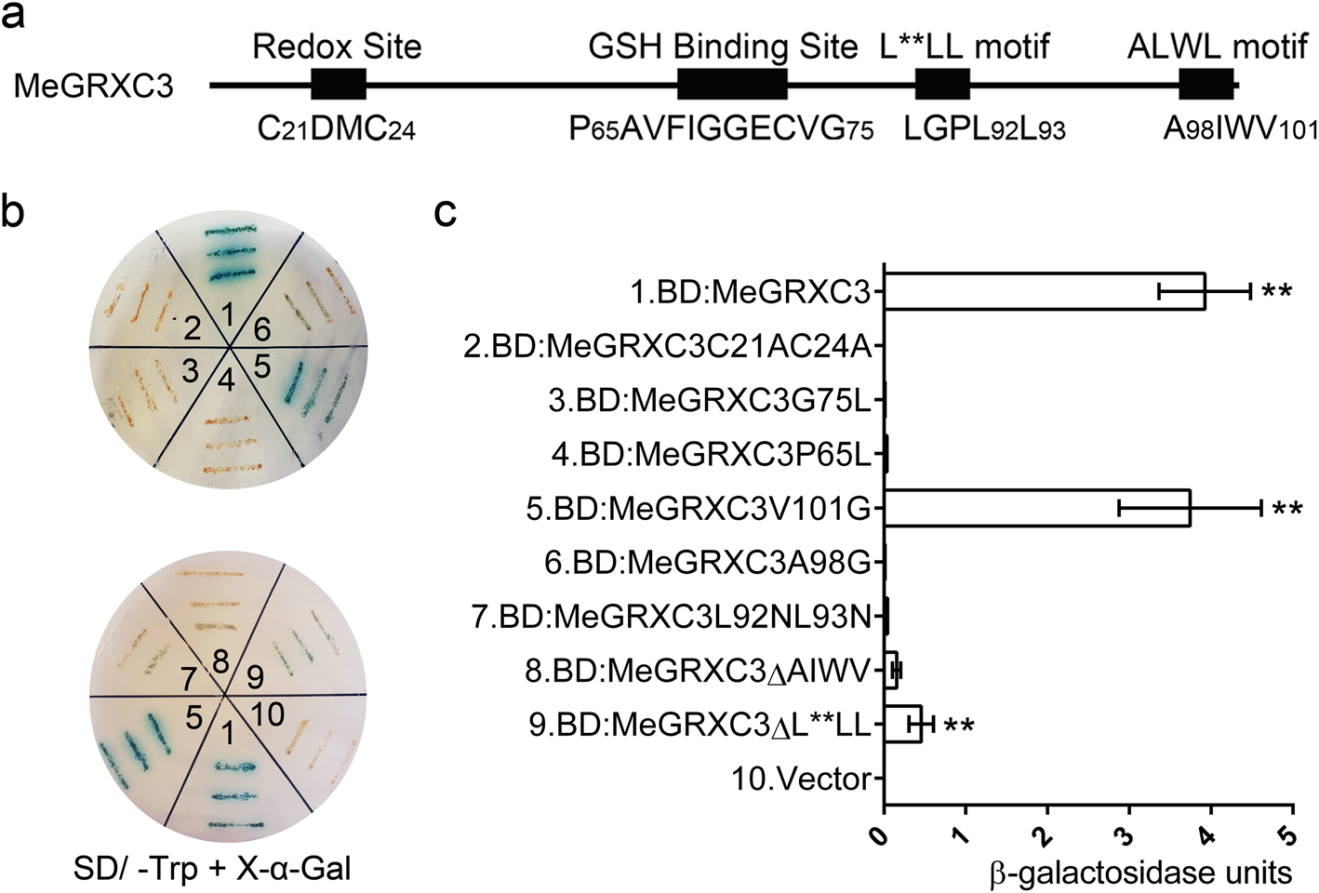
Conserved motifs are required for autonomous transactivation of MeGRXC3 in yeast. (**a**) Schematic diagram represents conserved motifs within MeGRXC3. Number indicates the position of amino acid. (**b**) Autonomous transactivation analysis of MeGRXC3 mutants in yeast. Number represents indicated DNA construct in (**c**). (c) *β*-galactosidase quantitative analysis of MeGRXC3 mutants in yeast. *pGBKT7* (Vector) was used as control. Error bars indicate mean ± SD (n = 5). ** *p* ≤ 0.01 (*Student’s* t-test)

The ability of modulating TGA transcription factors is indispensable for CC-type GRXs function in *Arabidopsis* [12, 21, 30]. The CCMC redox motif and GSH bind motif is required for GRXs redox activity [21]. The L**LL and ALWL motif in CC-type GRXs C terminus are critical for its TGA transcription factors modulation [21, 30]. We have found that four cassava drought-responsive CC-type GRXs including MeGRXC3 show transcriptional activation ability in yeast (Fig. 1). According to conserved motifs within MeGRXC3 (Fig. 6a), we performed mutant on each motif and created a series of MeGRXC3 mutants, which were fused to GAL4 DNA binding domain, and transformed into yeast strain Y187 respectively. When the GSH binding motif has been mutated (P65L or G75L) caused loss of transcriptional activation ability (Fig. 6b, c). Moreover, mutation in the C-terminal L**LL motif (L92N and L93N) also resulted in transcriptional activation ability loss (Fig. 6b, c). However, mutation of the fourth amino acid in the C-terminal ALWL motif (V101G) did not affect transcriptional activation ability (Fig. 6b, c). While mutation of the first amino acid in the C-terminal ALWL motif (A98G) resulted in transcriptional activation ability loss (Fig. 6b, c). Furthermore, the CCMC motif of CC-type GRXs is required for its redox activity. Mutation of this motif (C21ADMC24A) also resulted in loss of transcriptional activation ability (Fig. 6b, c). Together, the results suggest that all the conserved motifs are required for the transcriptional activation ability of MeGRXC3 in yeast.

### Conserved motifs are indispensable for MeGRXC3 function in the nucleus

**Fig. 7:**
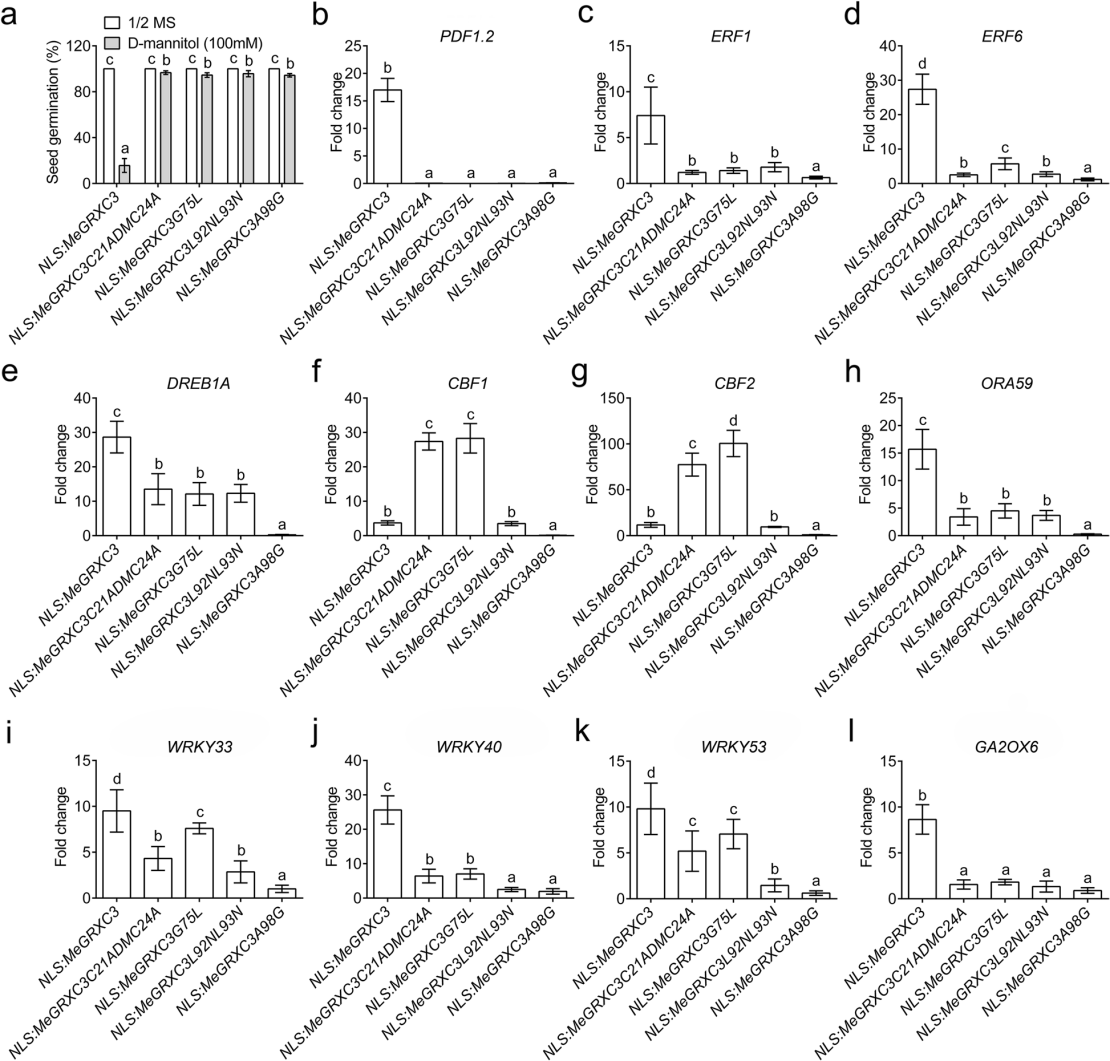
Conserved motifs are required for nuclear activity of MeGRXC3 in transgenic *Arabidopsis*. (**a**) Effects of mannitol stress on germination rate of *NLS:MeGRXC3* mutants overexpressed *Arabidopsis*. Seeds of three independent homozygote lines sown on 1/2 MS medium supplemented with 0 mM, or 100 mM D-mannitol respectively, incubated at 22 °C for 14 days. Different letters represent a significant difference at *p* < 0.05 (Duncan’s multiple range tests). Error bars indicate mean ± SD (*n* = 3). (**b**-**i**) Gene expression analysis in *NLS:MeGRXC3* mutants overexpressed *Arabidopsis*. Expression levels of selecting genes were normalized against wild type *Arabidopsis* (Col-0). Different letters represent a significant difference at *p* < 0.05 (Duncan’s multiple range tests). Error bars indicate mean ± SD (*n* = 3)

To truly understand the nuclear contribution of MeGRXC3 function *in planta*, we expressed a series of *NLS:MeGRXC3:GFP* mutant constructs, driven by the *35S* promoter in *Arabidopsis*. Herein, mutation of A98G in the C-terminal ALWL motif and mutation of L92NL93N in the L**LL motif in NLS:MeGRXC3:GFP fusion protein resulted a dramatic recovery in seed germination under 100 mM mannitol treatment (Fig. S6; Fig. 7a), suggesting that MeGRXC3 functions in the nucleus likely dependent on interaction and modification of TGA transcription factors. Redox site (C_21_CMC_24_) and GSH binding site (P_65_*********G_75_) are required for the redox activity of CC-type GRX. Substitution mutants of CCMC motif C21ADMC24A and GSH binding motif G75L of MeGRXC3 were fused to NLS at N-terminus and GFP at C-terminus respectively. Likewise, substitution mutants of these two motifs also caused a striking recovery in seed germination under mannitol treatment (Fig. S6; Fig. 7a). These results indicate that redox activity is indispensable for MeGRXC3 function in regulating mannitol-induced osmotic stress response in transgenic *Arabidopsis*.

We therefore analyzed the gene expression alteration by mutation of MeGRXC3 conserved motifs in the abovementioned transgenic *Arabidopsis* plants under normal conditions. Nuclear overexpression of *MeGRXC3* (*NLS:MeGRXC3*) dramatically enhanced the expression of *PDF1.2, ERF1, ERF6, ORA59, DREB2A, WRKY33, WRKY40, WRKY53,* and *GA2OX6* in transgenic *Arabidopsis* (Fig. 7b-i). However, *NLS:MeGRXC3* induced expression enhancement of these seven gene was obviously reduced by substitution mutations in MeGRXC3 conserved motifs, especially by L92NL93N and A98G mutations (Fig. 7b-i). These results imply these conserved motifs are required for the function of *MeGRXC3* in the nucleus.

## Discussion

CC-type GRX is a land plant-specific GRX subgroup that participates in organ development and stress responses through interaction with TGA transcription factors. Recently, several CC-type GRXs have been intensively studied for their role in plant abiotic stress response and phytohormone signalling [5, 15, 16, 32, 33]. We have found that six CC-type GRXs, including *MeGRXC3, MeGRXC4, MeGRXC7, MeGRXC14, MeGRXC15,* and *MeGRXC18* were induced by drought stress and exogenous ABA treatments in leaves of cassava cultivars [8]. This suggesting that CC-type GRXs regulated drought response probably in an ABA-dependent pathway. However, it is difficult to analyze all these drought-responsive CC-type GRXs in transgenic cassava. We need criteria for choosing candidate genes that should be further investigated. Therefore, we characterized these cassava genes in yeast and *Arabidopsis* to investigate the potential regulatory roles of them. Cassava is very different from *Arabidopsis*, the results cannot be used to determine biological function of cassava CC-type GRX, but it provides clues for further analysis of these genes in transgenic cassava.

Fusion of *Arabidopsis* ROXYs to GAL4 BD shows no autonomous transactivation in yeast [12, 21, 30]. By contrast, in our study, BD-MeGRXC3, BD-MeGRXC7, BD-MeGRXC14, and BD-MeGRXC15 exhibited strong autonomous transactivation activity in yeast (Fig. 1), indicating that MeGRXC3 could recruit transcription factor in yeast nucleus and generate a complex protein like GAL4BD-MeGRX-TF (Activation Domain). Thus, the recombination structure was able to function as a transcription factor promoting the transcription of reporter gene in yeast strain Y187. However, MeGRXC4 and MeGRXC18 did not show autonomous transactivation activity in yeast (Fig. 1). These results suggest these CC-type GRXs may play different roles in cassava drought response. Therefore, we produced transgenic *Arabidopsis* that overexpressing *MeGRXC3*, *MeGRXC4*, *MeGRXC15*, and *MeGRXC18* respectively, to identify whether they have different functions in plant.

To evaluate abiotic stress tolerance of transgenic plants, researchers commonly use in vitro setups in which different inhibitory supplements are added to the culture medium. For example, mannitol and polyethylene glycol (PEG) are frequently applied supplements to induce stress to the plant. Our data showed that mannitol treatment dramatically inhibited seed germination of *MeGRXC3*-OE transgenic *Arabidopsis*, but did not affect that of *MeGRXC4*-OE, *MeGRXC15*-OE, and *MeGRXC18*-OE transgenic *Arabidopsis* (Fig. 2; Fig. S1; Table 1). In parallel, overexpression of *MeGRXC3* enhanced mannitol-induced seedling growth inhibition in transgenic *Arabidopsis* (Fig. 2). These indicating that cassava drought-responsive CC-type GRXs really have different functions in plant. Mannitol can result in activation of stress-responsive genes, such as several ETHYLENE RESPONSE FACTORs including *ERF1, ERF6* in *Arabidopsis* [29, 34]. Here, *MeGRXC3* overexpression resulted in a significant up-regulation of *ERF6* in transgenic *Arabidopsis* (Fig. 3). In *Arabidopsis*, Overexpression of *ERF6* caused extreme mannitol-induced growth inhibition, which directly activates many stress-responsive and transcriptional regulation genes such as *WRKY33* and *GA2OX6* [35]. Here, these two genes were also up-regulated by overexpression of *MeGRXC3* in transgenic *Arabidopsis* (Fig. 3). Therefore, we propose the hypothesis that *MeGRXC3* negatively regulates mannitol tolerance by up-regulating *ERF6* in transgenic *Arabidopsis*. *ERF6* could regulate expression of ROS-induced gene such as *RBOHs*, *PRX, APX4,* and *CATs*, play an important role in ROS signaling pathway in *Arabidopsis* [36–40]. ROS is the most essential signaling of drought induced leaf abscission, which might be regulated by several ERF transcription factors in cassava cultivars [41, 42]. Compared with wild type, *MeGRXC3*-OE transgenic *Arabidopsis* seedlings accumulated more ROS (Fig. S7). Together, it implies the possibility of *MeGRXC3* involving drought induced leaf abscission in cassava through regulating ERF transcription factors. Expression of *ERF6* could be induced by exogenous ROS treatment [39], however, it is not clear whether the endogenous ROS increment up-regulate *ERF6* expression in *MeGRXC3*-OE transgenic *Arabidopsis*.

In *Arabidopsis*, ROXY19/GRX480 repressing the JA/ET pathway by negatively modified TGA2 [21]. TGA2 is a bZIP transcription factor, could recognize TGACG element in the promoter of many stress-inducible genes [25, 43]. Ectopically expressed *ROXY19/GRX480* repressed many genes which contained TGACG element in the promoter [22]. However, overexpression of *MeGRXC3* in *Arabidopsis* enhanced the expression of several transcription factors involved in JA/ET pathway, such as *PDF1.2*, *ERF6*, *ORA59*, *WRKY33*, and *WRKY53* (Fig. 3). Expression of *PDF1.2* is regulated by ethylene responsive transcription factor *ORA59*, *ROXY19/GRX480* suppressed *ORA59* promoter activity through negatively modifying TGA2 therefore repressed expression of *PDF1.2* [21]. On the contrary, *MeGRXC3* overexpression dramatically up-regulated *ORA59* and *PDF1.2* (Fig. 3). It indicating that MeGRXC3 may positively modify TGA2 in transgenic *Arabidopsis*. This result is consistent with MeGRXC3 showing transcription activation ability in yeast (Fig. 1). In addition, we have found that *MeGRXC3* was induced by exogenous ABA application in cassava cultivars [8]. Like MeGRXC15, MeGRXC3 also interacted with *Arabidopsis* TGA2 and TGA5 in the nucleus (Fig. 4). Together, it can be concluded that *MeGRXC3* involving crosstalk between ABA and JA/ET signalling pathways through positively modifying TGA2.

The nuclear interaction with TGA factors is required for ROXY1 function in petal development of *Arabidopsis* [12]. Likewise, eliminated nuclear localization of MeGRXC3 failed to result mannitol-induced germination and growth inhibition in transgenic *Arabidopsis* (Fig. 5). This indicates that nuclear localization is required for function of MeGRXC3 in transgenic *Arabidopsis* under mannitol stress. Additionally, the redox site is required for disulfide reductase activity of CC-type GRXs and GSH is the cofactor for the reduction reaction. Substitution mutants in redox site (C_21_DMC_24_) and GSH (P_65_*********G_75_) binding site of MeGRXC3 caused autonomous transactivation activity loss in yeast (Fig. 6), and abolished mannitol hypersensitivity in transgenic *Arabidopsis* (Fig. 7a). Furthermore, these two substitution mutants significantly altered the regulation of MeGRXC3 on expression of *ERF6* and *ORA59* (Fig. 7b, d, h). This suggests that the redox activity of MeGRXC3 is essential for the regulation of the target transcription factor. The C-terminal L**LL and ALWL motif in CC-type GRXs are necessary for their interaction with TGA transcription factors [12, 21, 30]. And the ALWL motif is required for ROXY19/GRX480 repressing the expression of *PDF1.2* and *ORA59* by interaction with TGA2 in *Arabidopsis* [21]. Mutations or deletion of the C-terminal L**LL motif (LGPL_92_L _93_) or ALWL motif (A_98_IWV) of MeGRXC3 also resulted in alterations of autonomous transactivation activity in yeast (Fig. 6), abolishment of mannitol hypersensitivity in transgenic *Arabidopsis* (Fig. 7a), and alterations of stress-related genes regulation in nucleus (Fig. 7b-i), indicating that the interaction with TGA transcription factor is required for the functions of MeGRXC3. Together, our data implying that MeGRXC3 is able to recruit and positively modified a TGA transcription factor in plant.

## Conclusions

CC-type GRXs play important roles with TGA transcription factors in the regulation of organ development, seed germination, defense pathway, nitrate metabolism, and abiotic stress responses. We have identified six drought-responsive CC-type GRXs from cassava cultivars, however, the molecular functions of these genes are still unclear. This study demonstrates that a cassava CC-type GRX, namely *MeGRXC3*, regulates mannitol-induced osmotic stress tolerance by nuclear interaction with TGA transcription factors and positively regulating several stress-related transcription factors including *ERF6* and *ORA59*.

## Methods

### Plant materials

Seeds of *Arabidopsis thaliana* ecotype Columbia-0 (Col-0, ABRC stock number CS60000) was obtained from ABRC and kept in our lab (Institute of Tropical Biosciences and Biotechnology, Chinese Academy of Tropical Agricultural Sciences, Haikou, China). Experimental research on all plants complied with institutional and national guidelines. *Arabidopsis* and tobacco plants were grown in greenhouse at the Institute of Tropical Biosciences and Biotechnology (Haikou, China). The plants were grown under 12 h light/12 h dark at 20–23 °C until the primary inflorescence was 5-15 cm tall and a secondary inflorescence appeared at the rosette. *Arabidopsis* transformation was achieved using the floral dip method [44] with *A. tumefaciens* strain *LBA4404* carrying the appropriate DNA constructs.

### Transactivation analysis in yeast

The *MeGRXC3, MeGRXC4, MeGRXC7, MeGRXC14, MeGRXC15,* and *MeGRXC18* were in frame fused to the GAL4 binding domain (BD) in *pGBKT7* (Clontech) respectively. The stop-codon-less coding sequences of *MeGRXC3* mutants were also fused in-frame to the DNA binding domain of GAL4 BD in *pGBKT7*. The resulted constructs were confirmed by sequencing and transferred into yeast strain *Y187* (Clontech). Yeast cells were selected on SD/−Trp medium and positive colonies were checked by PCR using gene specific primers. Three yeast colonies harboring the indicated plasmid were incubated at 30 °C on SD/−Trp medium containing 20 μg/mL X-α-gal until blue colonies were formed.

### Seed germination assays of transgenic *Arabidopsis*

For the germination assays, seeds of each line were surface sterilized, sown on solid agar medium plates (1/2 MS, pH 5.7, and 0.7% phytagel) with D-mannitol (0 mM, 100 mM, or 200 mM). Seeds were incubated in the dark at 4 °C for 48 h, and then incubated in 8 h/16 h light/dark growing chamber at 22 °C. Germination was judged by the protrusion of the radicle and the germination rate was scored as the percentage at 14 days after sowing. For each germination assay, the offspring of three independent homozygous lines were used, and at least three biological replicate experiments were performed.

### Mannitol tolerance assays of transgenic *Arabidopsis* seedling

To study the response of transgenic *Arabidopsis* seedling to mannitol stress, seeds were sown on 1/2 MS medium, then postgermination seedlings were transferred to 1/2 MS medium containing with 0 mM or 100mMD-mannitol at 7 days after sowing. Seedlings were incubated in 8 h/16 h light/dark growing chamber at 22 °C for 14 days. Biomass of treating seedlings was measured as total dry weight. The results were shown as percentage, which biomass of wild type seedlings on 1/2 MS medium containing with 0 mM D-mannitol was indicated as 100%.

### Quantitative real-time PCR (qPCR) analysis

Total RNA was isolated from *Arabidopsis* leaves using an RNAprep Pure Plant Kit (TIANGEN). cDNA synthesis was performed using FastQuant RT Kits (TIANGEN). Gene expression analysis in *Arabidopsis* was performed by qPCR with gene-specific primers (Table S1). All qPCR reactions were carried out in triplicate, with SYBR® Premix Ex Taq™ II Kit (Takara) on a StepOne™ Real-Time PCR system (Applied Biosystems). The comparative ΔΔCT method was employed to evaluate amplified product quantities in the samples.

### Protein subcellular localization

Leaves from 4-week-old *Nicothiana benthamiana* plants were transformed by infiltration using a 5-mL syringe (without needle) to transfer *Agrobacterium* cells (OD_600_ = 1.2) harboring appropriate DNA constructs. After 3 days, infiltrated *N. benthamiana* leaves were examined for reconstitution of GFP fluorescence by a confocal laser scanning microscope (Olympus FluoView FV1100).

### Yeast two-hybrid assay

For screen the interaction proteins of MeGRXC3, a yeast two-hybrid assay has been performed in yeast strain Y2HGold based on the Matchmaker® Gold Yeast Two-Hybrid System User Manual (Clontech). DNA construct of *MeGRXC3P65L:pGBKT7* was used as bait. The cDNA sequences of *Arabidopsis TGA1, TGA2, TGA4, TGA5*, and *TGA7* were introduced into the *pGADT7*, in frame fused to GAL4 activate domain (AD). All constructs were pairwise co-transformed into yeast strain Y2HGold. The presence of transgenes was confirmed by growth on DDO (SD/−Leu/−Trp) plates. Interactions between two proteins were confirmed by growth on QDO/X/A (SD/−Ade/−His/−Leu/−Trp with 40 μg/mL X-alpha-Gal and 200 ng/mL Aureobasidin A).

### Bimolecular fluorescence complementation

To confirm the interactions between MeGRXC3 and TGA factors, a bimolecular fluorescence complementation assay has been performed by tobacco transient system as previously report [45]. The full-length coding sequence without stop-codon of *MeGRXC3* was in frame fused to N- or C-terminus of the yellow fluorescent protein (YFP) fragment (YN/YC) respectively to produce *35S:MeGRXC3:YN:pBiFC* and *35S:MeGRXC3:YC:pBiFC.* The full-length coding sequence without stop-codon of *TGA2* and *TGA5* were in frame fused to YC or YN respectively to produce *35S:TGA2:YC:pBiFC, 35S:TGA5:YN:pBiFC, 35S: TGA2:YC:pBiFC,* and *35S: TGA5:YN:pBiFC.* The resulting constructs were then introduced into *A. tumefaciens LBA4404* strains. Then the assays were performed as the method of protein subcellular localization described.

### Mutation of MeGRXC3

Nuclear localization signal sequence (PKKKRKV) from the SV40 large T antigen was fused to the N-terminus of MeGRXC3:GFP by PCR method to create *NLS:MeGRXC3*:*GFP* as described in reference [12]. Three folds of GFP (3 × GFP) DNA was synthesized and fused to C-terminus of MeGRXC3 to create *MeGRXC3:3 × GFP* as described in reference [12]. Conserved motifs in MeGRXC3 were mutated by site-directed mutation method to make NLS:MeGRXC3 mutants. The C_21_DMC_24_ motif was modified to A_21_DMA_24_. The P_65_ was replaced by L_65_ as well as G_75_ was replaced by L_75_ in GHS binding motif respectively. The C-terminal LGPL_92_L_93_ motif was replaced by LGPN_92_N_93_. The very C-terminal motif A_98_IWV_101_ motif was replaced by G_98_IWI and AIWV_101_, respectively. *β*-galactosidase quantitative analysis was performed in Y187 strain that harboring respective *MeGRXC3mutant:pGBKT7* construct according to Matchmarker® Gold Yeast Two-Hybrid System User Manual (Clontech).

### Accession numbers

The cDNA sequences of cassava CC-type GRXs were downloaded from the cassava genome database (*Manihot esculenta* v6.1), and cDNA sequences of *Arabidopsis* were downloaded from the *Arabidopsis thaliana* TAIR10 as the accession numbers indicated in Phytozome (https://phytozome.jgi.doe.gov/pz/portal.html). Gene accession numbers were listed as following: *MeGRXC3* (*Manse. 01G215000.1*), *MeGRXC4* (*Manes. 01G215100.1*), *MeGRXC7* (*Manes. 05G066700.1*), *MeGRXC14* (*Manes. 15G015500.1*), *MeGRXC15* (*Manes.15G015600.1*), *MeGRXC18* (*Manes. 17G050200.1*), *PDF1.2* (*AT5G44420.1*), *ERF1* (*AT3G23240.1*), *ERF6* (*AT4G17490.1*), *ORA59* (*AT1G06160*), *DREB2A* (*AT5G05410*), *CBF1* (*AT4G25490*), *CBF2* (*AT4G25470*), *WRKY33* (*AT2G38470.1*), *WRKY40* (*AT1G80840.1*), *WRKY53* (*AT4G23810.1*), *GA2OX6* (*AT1G02400.1*), *ACT1* (*AT3G53750.1*), *TGA1* (*AT5G65210*), *TGA2* (*AT5G06950*), *TGA5* (*AT5G06960*), *TGA4* (*AT5G10030*), *TGA7* (*AT1G77920*).

## Supplementary Information

**Additional file 1: Table S1.** The list of qPCR Primers.

**Additional file 2: Figure S1.** Identification of *MeGRXC3*-OE, *MeGRXC4*-OE, *MeGRXC15*-OE and *MeGRXC18*-OE *Arabidopsis*.

**Additional file 3: Figure S2.** Seed germination assay of *MeGRXC3-*OE, *MeGRXC4*-OE, *MeGRXC15*-OE, and *MeGRXC18*-OE on 1/2 MS containing with 100 mM or 200 mM D-mannitol.

**Additional file 4: Figure S3.** Seedling growth inhibition assay of *MeGRXC3*-OE transgenic *Arabidopsis* under 100 mM D-mannitol treatment.

**Additional file 5: Figure S4.** Seed germination assay of *MeGRXC3:3 × GFP* and *NLS:MeGRXC3* transgenic *Arabidopsis* under 100 mM D-mannitol treatment.

**Additional file 6: Figure S5.** Seedling growth inhibition assay of *MeGRXC3:3 × GFP* transgenic *Arabidopsis* under 200 mM D-mannitol treatment.

**Additional file 7: Figure S6.** Seed germination assay of *NLS:MeGRXC3C21ADMC24A, NLS:MeGRXC3G75L, NLS:MeGRXC3L92NL93N,* and *NLS:MeGRXC3A98G* transgenic *Arabidopsis*.

**Additional file 8: Figure S7.** Diaminobezidin (DAB) staining of *MeGRXC3*-OE and wild type *Arabidopsis* seedlings.

## Data Availability

Data sharing is not applicable to this article as no datasets were generated or analyzed during the current study.

## Abbreviations

GRX: Glutaredoxin
AD: Activation domain
BD: Binding domain
JA: Jasmonic acid
ET: Ethylene
ABA: Abscisic acid
GA: Gibberellin acid
ROS: Reactive oxygen species
GFP: Green fluorescent protein
YFP: Yellow fluorescent protein
NLS: Nuclear localization signal
qPCR: Quantitative real-time polymerase chain reaction
MV: Methyl viologen
HL: High light
