# Supplementary material for: Role of cassava CC-type glutaredoxin MeGRXC3 in regulating sensitivity to mannitol-induced osmotic stress dependent on its nuclear activity
Source: BMC Plant Biol. 2022 Jan 20;22:41. doi: 10.1186/s12870-022-03433-y (PMC8772167; doi:10.1186/s12870-022-03433-y)
Supplement: Supplementary file 2 — Additional file 2: Figure S1. Identification of MeGRXC3-OE, MeGRXC4-OE, MeGRXC15-OE and MeGRXC18-OE Arabidopsis. [file 12870_2022_3433_MOESM2_ESM.pdf]

Figure S1

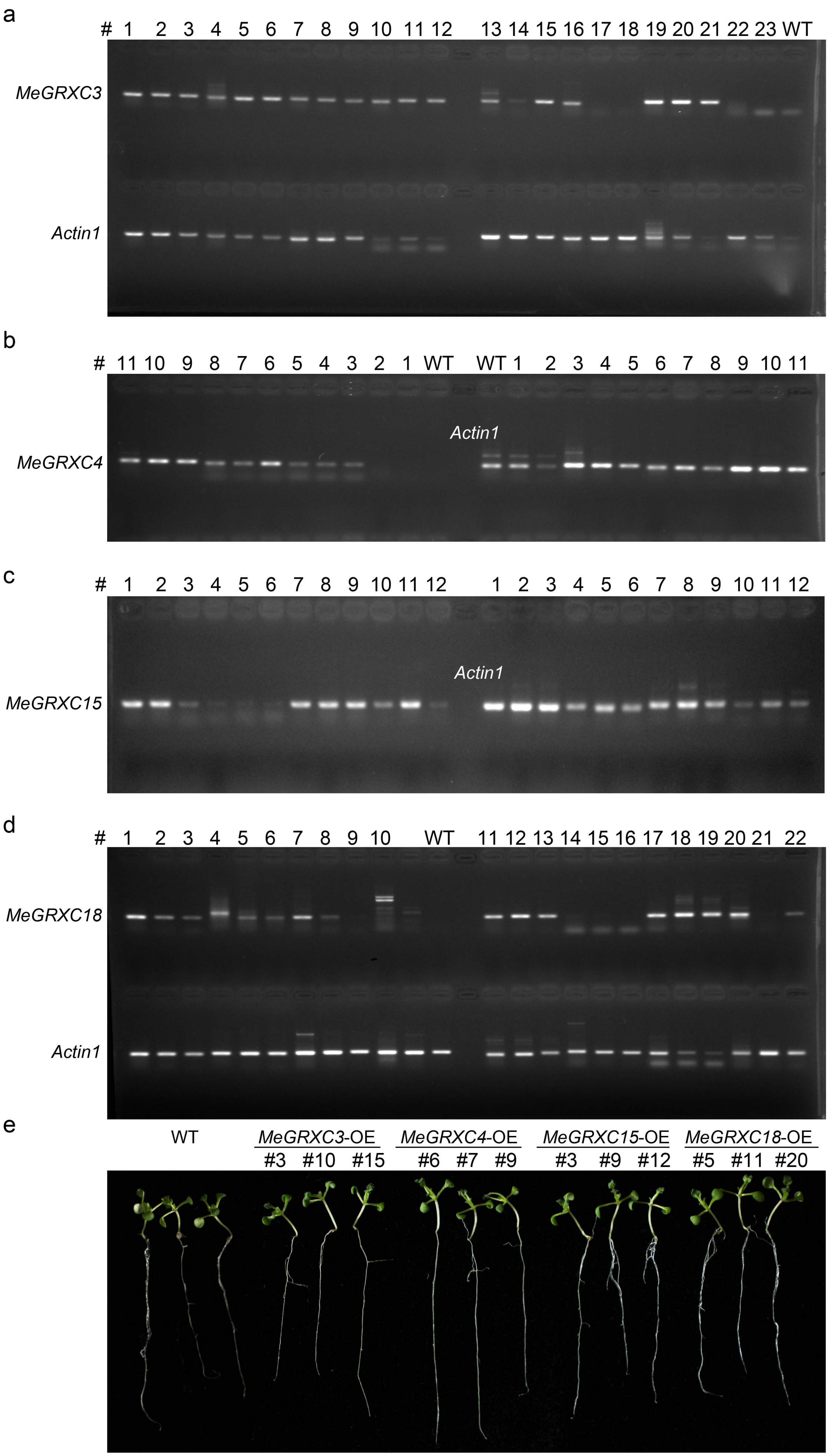

a
1 2 3 4 5 6 7 8 9 10 11 12 13 14 15 16 17 18 19 20 21 22 23 WT
MeGRXC3
Actin1
b
11 10 9 8 7 6 5 4 3 2 1 WT WT 1 2 3 4 5 6 7 8 9 10 11
MeGRXC4
Actin1
c
1 2 3 4 5 6 7 8 9 10 11 12 1 2 3 4 5 6 7 8 9 10 11 12
MeGRXC15
Actin1
d
1 2 3 4 5 6 7 8 9 10 WT 11 12 13 14 15 16 17 18 19 20 21 22
MeGRXC18
Actin1
e
WT
MeGRXC3-OE
#3 #10 #15
MeGRXC4-OE
#6 #7 #9
MeGRXC15-OE
#3 #9 #12
MeGRXC18-OE
#5 #11 #20

Figure S1. Identification of *MeGRXC3*-OE, *MeGRXC4*-OE, *MeGRXC15*-OE and *MeGRXC18*-OE *Arabidopsis*. a. RT-PCR identification of *MeGRXC3*-OE transgenic *Arabidopsis*. b. RT-PCR identification of *MeGRXC4*-OE transgenic *Arabidopsis*. c. RT-PCR identification of *MeGRXC15*-OE transgenic *Arabidopsis*. d. RT-PCR identification of *MeGRXC18*-OE transgenic *Arabidopsis*. e. Seedlings of *MeGRXC3*-OE, *MeGRXC4*-OE, *MeGRXC15*-OE, and *MeGRXC18*-OE transgenic *Arabidopsis* and wild type *Arabidopsis*. WT: wild type. Numbers indicate transgenic line. *Actin1* was used as house keep gene in RT-PCR analysis.
