# Supplementary material for: Role of cassava CC-type glutaredoxin MeGRXC3 in regulating sensitivity to mannitol-induced osmotic stress dependent on its nuclear activity
Source: BMC Plant Biol. 2022 Jan 20;22:41. doi: 10.1186/s12870-022-03433-y (PMC8772167; doi:10.1186/s12870-022-03433-y)
Supplement: Supplementary file 3 — Additional file 3: Figure S2. Seed germination assay of MeGRXC3-OE, MeGRXC4-OE, MeGRXC15-OE, and MeGRXC18-OE on 1/2 MS containing with 100 mM or 200 mM D-mannitol. [file 12870_2022_3433_MOESM3_ESM.pdf]

Figure S2

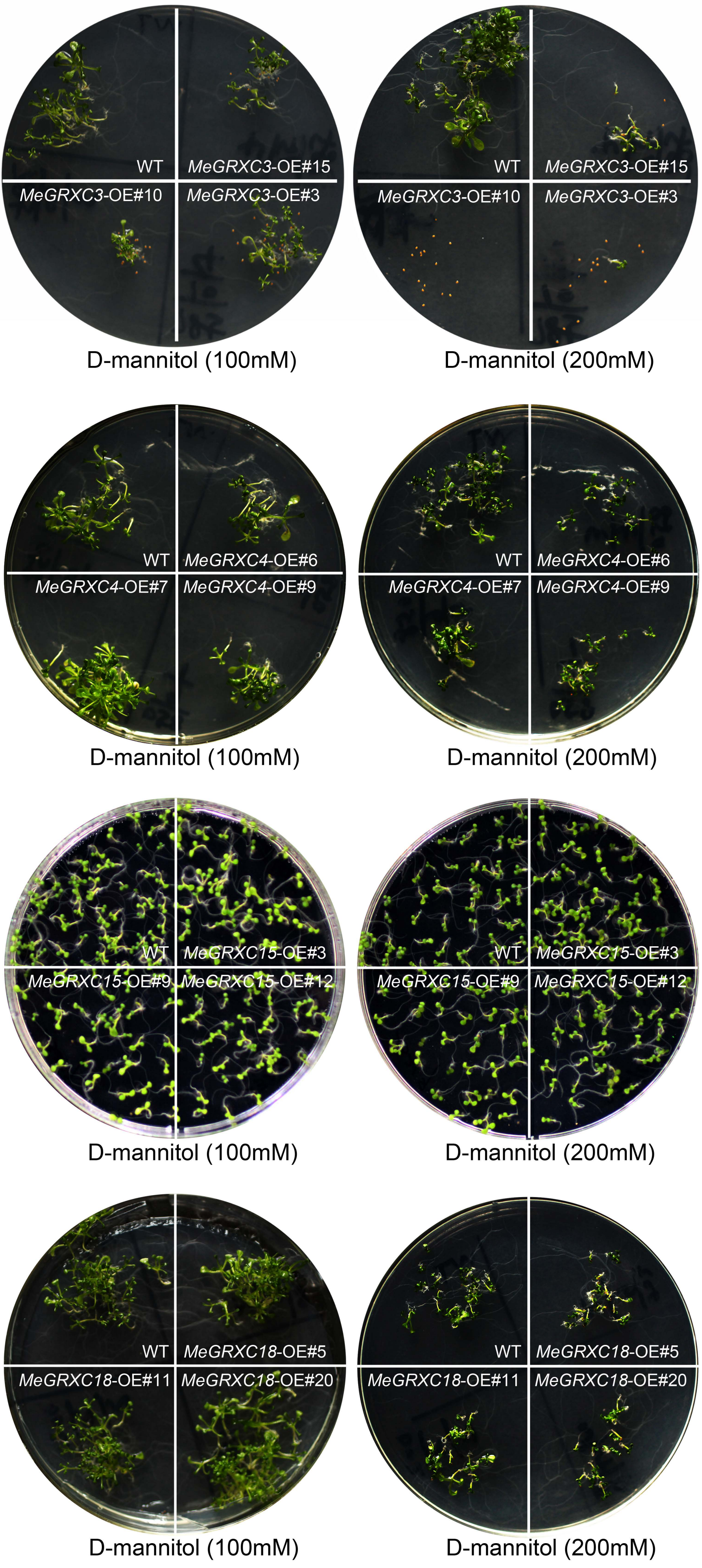
WT
MeGRXC3-OE#15
MeGRXC3-OE#10
MeGRXC3-OE#3
D-mannitol (100mM)
WT
MeGRXC3-OE#15
MeGRXC3-OE#10
MeGRXC3-OE#3
D-mannitol (200mM)
WT
MeGRXC4-OE#6
MeGRXC4-OE#7
MeGRXC4-OE#9
D-mannitol (100mM)
WT
MeGRXC4-OE#6
MeGRXC4-OE#7
MeGRXC4-OE#9
D-mannitol (200mM)
WT
MeGRXC15-OE#3
MeGRXC15-OE#9
MeGRXC15-OE#12
D-mannitol (100mM)
WT
MeGRXC15-OE#3
MeGRXC15-OE#9
MeGRXC15-OE#12
D-mannitol (200mM)
WT
MeGRXC18-OE#5
MeGRXC18-OE#11
MeGRXC18-OE#20
D-mannitol (100mM)
WT
MeGRXC18-OE#5
MeGRXC18-OE#11
MeGRXC18-OE#20
D-mannitol (200mM)

Figure S2. Seed germination assay of *MeGRXC3*-OE, *MeGRXC4*-OE, *MeGRXC15*-OE, and *MeGRXC18*-OE on 1/2 MS containing with 100mM or 200mM D-mannitol. Seeds of three independent homozygote lines per construct sown on 1/2 MS medium supplemented with 100mM or 200mM D-mannitol respectively, incubated at 22℃ for 14 days.
