# Supplementary material for: Role of cassava CC-type glutaredoxin MeGRXC3 in regulating sensitivity to mannitol-induced osmotic stress dependent on its nuclear activity
Source: BMC Plant Biol. 2022 Jan 20;22:41. doi: 10.1186/s12870-022-03433-y (PMC8772167; doi:10.1186/s12870-022-03433-y)
Supplement: Supplementary file 4 — Additional file 4: Figure S3. Seedling growth inhibition assay of MeGRXC3-OE transgenic Arabidopsis under 100 mM D-mannitol treatment. [file 12870_2022_3433_MOESM4_ESM.pdf]

Figure S3

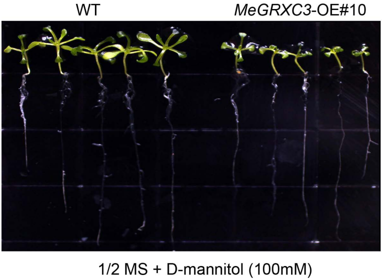

Figure S3. Seedling growth inhibition assay of *MeGRXC3*-OE transgenic *Arabidopsis* under 100mM D-mannitol treatment. Seedlings grown on 1/2 MS medium were transferred to 1/2 MS supplemented with 0mM or 100mM D-mannitol at 7 days after sowing respectively, then incubated at 22°C for 14 days.
