# Supplementary material for: Role of cassava CC-type glutaredoxin MeGRXC3 in regulating sensitivity to mannitol-induced osmotic stress dependent on its nuclear activity
Source: BMC Plant Biol. 2022 Jan 20;22:41. doi: 10.1186/s12870-022-03433-y (PMC8772167; doi:10.1186/s12870-022-03433-y)
Supplement: Supplementary file 5 — Additional file 5: Figure S4. Seed germination assay of MeGRXC3:3 × GFP and NLS:MeGRXC3 transgenic Arabidopsis under 100 mM D-mannitol treatment. [file 12870_2022_3433_MOESM5_ESM.pdf]

Figure S4

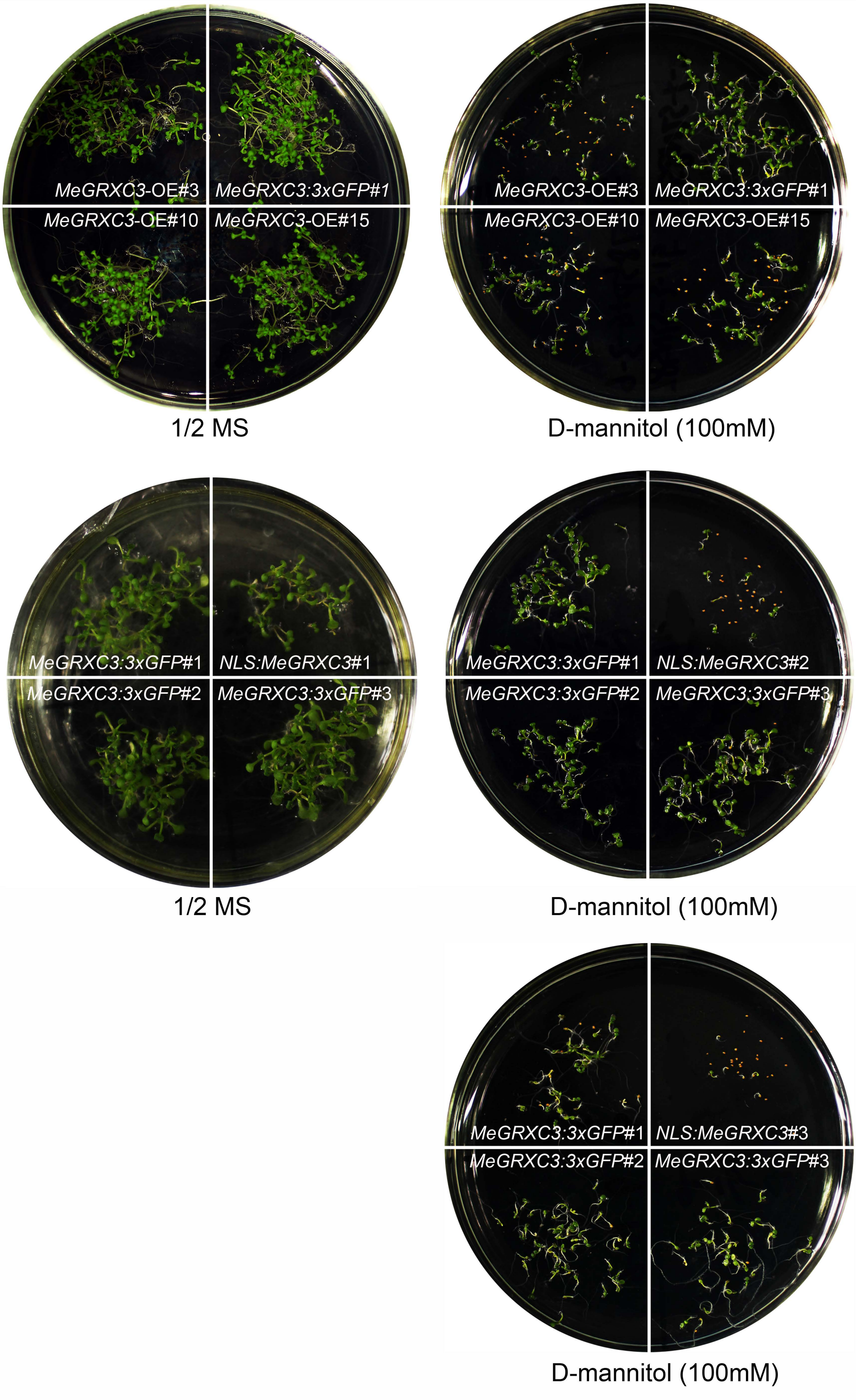
MeGRXC3-OE#3
MeGRXC3:3xGFP#1
MeGRXC3-OE#10
MeGRXC3-OE#15
1/2 MS
MeGRXC3-OE#3
MeGRXC3:3xGFP#1
MeGRXC3-OE#10
MeGRXC3-OE#15
D-mannitol (100mM)
MeGRXC3:3xGFP#1
NLS:MeGRXC3#1
MeGRXC3:3xGFP#2
MeGRXC3:3xGFP#3
1/2 MS
MeGRXC3:3xGFP#1
NLS:MeGRXC3#2
MeGRXC3:3xGFP#2
MeGRXC3:3xGFP#3
D-mannitol (100mM)
MeGRXC3:3xGFP#1
NLS:MeGRXC3#3
MeGRXC3:3xGFP#2
MeGRXC3:3xGFP#3
D-mannitol (100mM)

Figure S4. Seed germination assay of *MeGRXC3:3×GFP* and *NLS:MeGRXC3* transgenic *Arabidopsis* under 100mM D-mannitol treatment. Seeds of three independent homozygote lines sown on 1/2 MS medium supplemented with 0mM, or 100mM D-mannitol respectively, incubated at 22℃ for 14 days.
