# Supplementary material for: Role of cassava CC-type glutaredoxin MeGRXC3 in regulating sensitivity to mannitol-induced osmotic stress dependent on its nuclear activity
Source: BMC Plant Biol. 2022 Jan 20;22:41. doi: 10.1186/s12870-022-03433-y (PMC8772167; doi:10.1186/s12870-022-03433-y)
Supplement: Supplementary file 6 — Additional file 6: Figure S5. Seedling growth inhibition assay of MeGRXC3:3 × GFP transgenic Arabidopsis under 200 mM D-mannitol treatment. [file 12870_2022_3433_MOESM6_ESM.pdf]

Figure S5

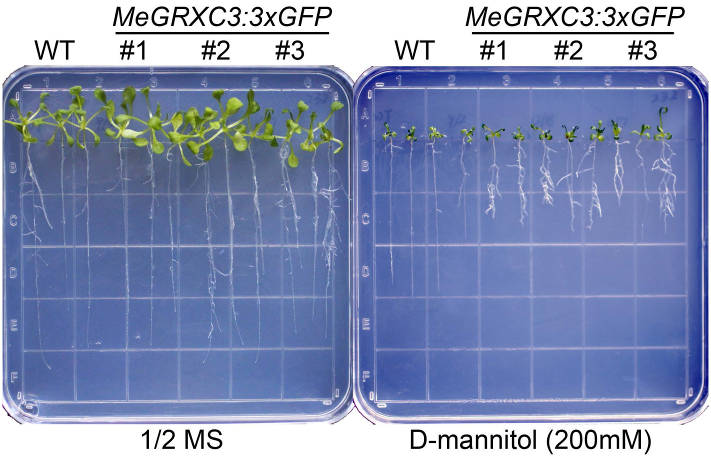
MeGRXC3:3xGFP
WT
#1
#2
#3
MeGRXC3:3xGFP
WT
#1
#2
#3
1/2 MS
D-mannitol (200mM)

Figure S5. Seedling growth inhibition assay of *MeGRXC3:3×GFP* transgenic *Arabidopsis* under 200mM D-mannitol treatment. Seedlings grown on 1/2 MS medium were transferred to 1/2 MS supplemented with 0mM or 200mM D-mannitol at 7 days after sowing respectively, then incubated at 22°C for 14 days.
