# Supplementary material for: Role of cassava CC-type glutaredoxin MeGRXC3 in regulating sensitivity to mannitol-induced osmotic stress dependent on its nuclear activity
Source: BMC Plant Biol. 2022 Jan 20;22:41. doi: 10.1186/s12870-022-03433-y (PMC8772167; doi:10.1186/s12870-022-03433-y)
Supplement: Supplementary file 7 — Additional file 7: Figure S6. Seed germination assay of NLS:MeGRXC3C21ADMC24A, NLS:MeGRXC3G75L, NLS:MeGRXC3L92NL93N, and NLS:MeGRXC3A98G transgenic Arabidopsis. [file 12870_2022_3433_MOESM7_ESM.pdf]

Figure S6

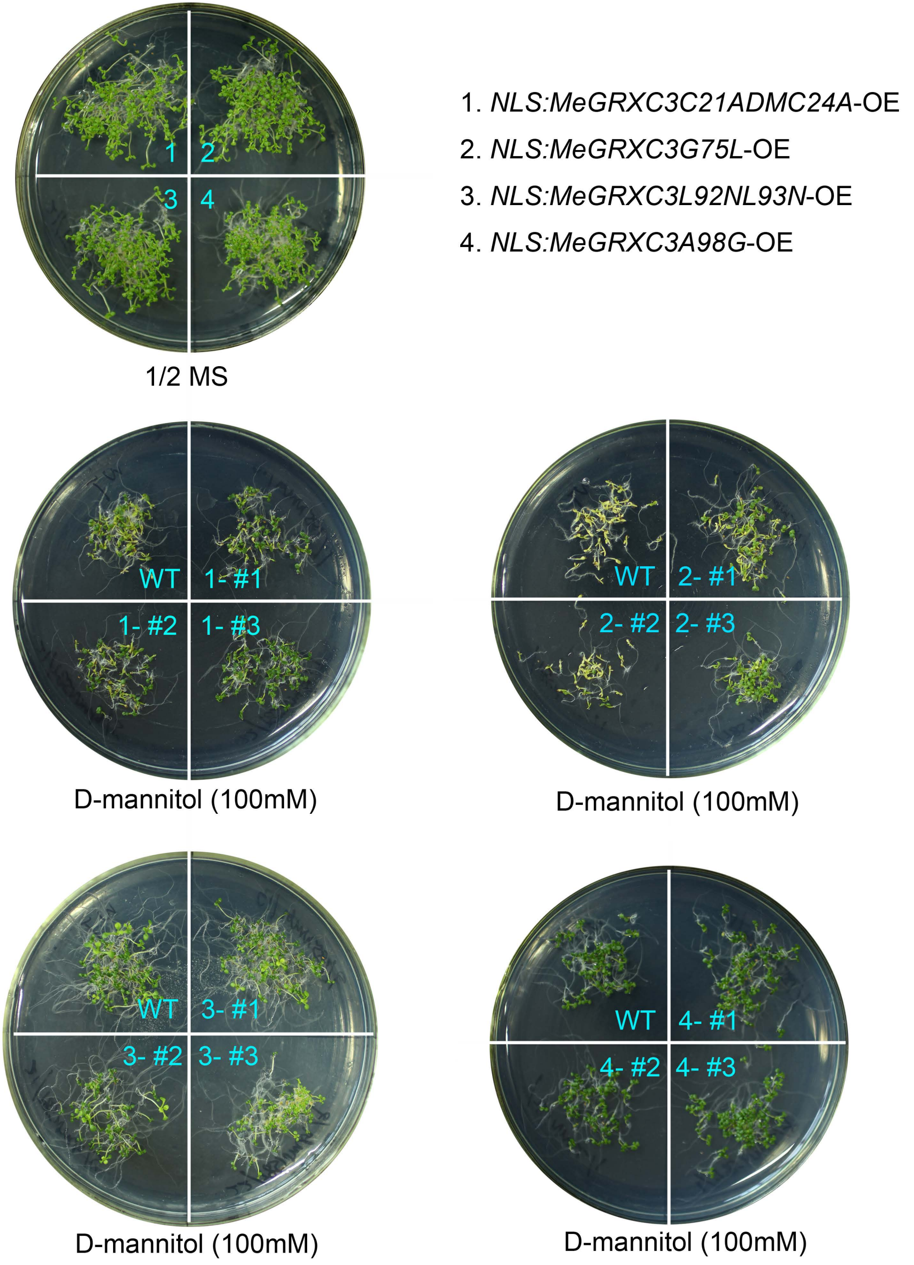

1. *NLS:MeGRXC3C21ADMC24A*-OE
2. *NLS:MeGRXC3G75L*-OE
3. *NLS:MeGRXC3L92NL93N*-OE
4. *NLS:MeGRXC3A98G*-OE
1 2 3 4
1/2 MS
WT 1- #1 1- #2 1- #3
D-mannitol (100mM)
WT 2- #1 2- #2 2- #3
D-mannitol (100mM)
WT 3- #1 3- #2 3- #3
D-mannitol (100mM)
WT 4- #1 4- #2 4- #3
D-mannitol (100mM)

Figure S6. Seed germination assay of *NLS:MeGRXC3C21ADMC24A, NLS:MeGRXC3G75L, NLS:MeGRXC3L92NL93N,* and *NLS:MeGRXC3A98G* transgenic *Arabidopsis*. Seeds of three independent homozygote lines per construct sown on 1/2 MS medium supplemented with 0mM or 100mM D-mannitol respectively, incubated at 22℃ for 14 days.
