# Supplementary material for: Role of cassava CC-type glutaredoxin MeGRXC3 in regulating sensitivity to mannitol-induced osmotic stress dependent on its nuclear activity
Source: BMC Plant Biol. 2022 Jan 20;22:41. doi: 10.1186/s12870-022-03433-y (PMC8772167; doi:10.1186/s12870-022-03433-y)
Supplement: Supplementary file 8 — Additional file 8: Figure S7. Diaminobezidin (DAB) staining of MeGRXC3-OE and wild type Arabidopsis seedlings. [file 12870_2022_3433_MOESM8_ESM.pdf]

Figure S7

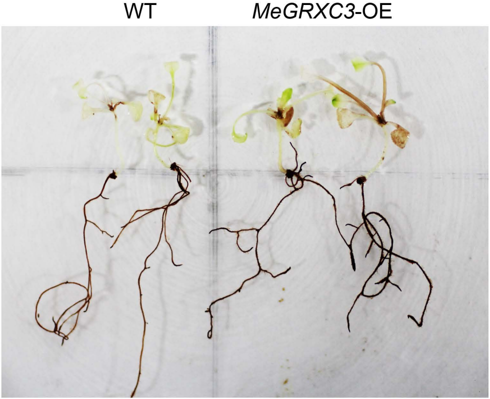

Figure S7. Diaminobezidin (DAB) staining of *MeGRXC3*-OE and wild type *Arabidopsis* seedlings. Seedlings (21 day after sowing) grown on 1/2 MS were infiltrated with 2 mL of DAB solution (1mg /mL DAB, pH 3.8) in Eppendorf tube for 12 hours. Then the seedlings were immersed in 95% (w/v) boiling ethanol for 10 min to decolorize the chloroplast.
